# An Experimental Approach for Selecting Appropriate Rodent Diets for Research Studies on Metabolic Disorders

**DOI:** 10.1155/2013/752870

**Published:** 2013-09-15

**Authors:** Suja Rani Sasidharan, Joshua Allan Joseph, Senthilkumar Anandakumar, Vijayabalaji Venkatesan, Chandrasekharan Nair Ariyattu Madhavan, Amit Agarwal

**Affiliations:** ^1^College of Veterinary and Animal Sciences, Mannuthy, Thrissur 680 651, India; ^2^R&D Centre, Natural Remedies, Plot No. 5B, Veerasandra Indl. Area, 19th K.M. Stone, Hosur Road, Electronic City, Bangalore, Karnataka 560 100, India

## Abstract

Diverse high energy diets have been utilized to precipitate obesity and related metabolic disorders in rodent models, though the dietary intervention has not absolutely been standardized. The present study established usage of a customized semipurified normal control diet (NCD) and high fat diet (HFD), for research studies on diet-induced metabolic disorders in albino rats. Male Wistar rats were fed with normal pellet diet (NPD) or customized NCDs I, II, III or HFDs I, II, III for 12 weeks and parameters, namely, body weight, visceral adiposity, serum triglycerides, cholesterol, and glucose were evaluated to select an appropriate NCD and HFD. The selected HFD was further evaluated for induction of fatty liver, whilst type 2 diabetes (T2D) induction was confirmed in HFD and streptozotocin (STZ) induced diabetes model in Wistar rats. Amongst different diets tested, NCD-I and HFD-I were selected, since NCD-I exhibited close resemblance to NPD, whereas HFD-I induced metabolic alterations, particularly obesity and dyslipidemia consistently. Moreover, HFD-I elevated terminal hepatic lipids, while HFD-I/STZ treatment augmented insulin resistance index and serum glucose levels significantly indicating effective induction of fatty liver and T2D, respectively. Therefore, customized semipurified NCD-I and HFD-I can be recommended for research studies on diet-induced metabolic disorders in albino Wistar rats.

## 1. Introduction

Nutritional science research during the 20th century has recognized diet as one of the potent environmental tools capable of changing the phenotype of an animal. One of the leading factors for the current global epidemic of obesity and its comorbidities such as insulin resistance, type 2 diabetes, dyslipidemia, hypertension, and nonalcoholic fatty liver disease is the western-style diet, which includes excessive intake of high energy foods. Consequently, diverse high energy diets have been utilized in rodent models to precipitate these metabolic disorders, though the dietary intervention is not yet absolutely standardized [1]. A careful choice of the species/strain and dietary intervention concomitant with adequate control over environmental variables will be of paramount importance in generating the repetitive data in diet-induced animal model studies [2]. 

While designing a diet-induced animal model for research studies on metabolic disorders, the composition of the control diet as well as high fat diet (HFD) deserves much attention. Laboratory animal diets have indeed been classified into three major categories: chows (cereal based diet), semipurified, and chemically defined diets which may exert significant independent effects on the measured phenotypes in any research protocol [3]. Chow diets containing plant-derived ingredients show variability in nutrient content and generally adhere to “closed” formula, as the exact amount of each ingredient is not revealed. It may also contain plant-derived phytoestrogens that can affect the progression of metabolic disease states [4]. On the other hand, semipurified diets, formulated from refined nutrient ingredients, can be intentionally modified to meet researcher's needs and contain negligible extraneous material, with very little batch to batch variation. The open source nature of semipurified diets allows researchers around the world to compare data from different studies. The third category is the chemically defined diets, made of chemically pure sources of amino acids, mono- or disaccharides, purified fatty acids or triglycerides, minerals, and the vitamins which represent the highest degree of control over nutrient concentrations. Unfortunately, they are not readily consumed by most species of laboratory animals and are usually too expensive for general use [3, 5]. Therefore, semipurified diets can be ideally considered for studies on metabolic disorders.

Due to increasing awareness for the need for nutritionally adequate semipurified diets, American Institute of Nutrition (AIN), an *ad hoc* committee which is formed to identify dietary standards for nutrition based research studies with laboratory rodents, has published AIN-93 rodent diet formula, which is again subclassified into AIN-93G and AIN-93 M as per growth and adult maintenance requirements, respectively [6]. The normal and high fat diets can be formulated based on AIN-93 diet, for which the quantity of individual ingredient may have to be modified. However, any dietary intervention needs proper standardization since the diet-induced phenotype varies distinctly among different study designs and also from lab to lab. 

Albeit utmost care is taken to ensure comparable genetic backgrounds and environmental conditions, while performing studies using diet-induced animal models, specific diet composition details are too often lacking in the literature, and also the diets used in the experiments are not well matched [7]. For instance, in many cases a chow diet has been used as a low fat “control” diet for comparison of a purified HFD or else; HFD is formulated by mixing fat enriched components, namely, lard, butter, beef tallow, and so forth, with the chow diet and thereby augmenting the confounding factors in the experiment [8]. While few of the international feed suppliers offer readymade semipurified diets with standardized recipes, its use is limited due to its high cost and unavailability of the diet in small batches from the suppliers. 

The present study was, therefore, conducted with the objective of identifying and selecting a customized, affordable semipurified normal control diet (NCD) and high fat diet (HFD), formulated based on AIN-93 rodent diet composition with minor modifications, for research studies on metabolic disorders.

## 2. Materials and Methods

### 2.1. Animals

Male albino Wistar rats (160–180 g body weight) of the present study were acclimatized for a week before experimentation and maintained at optimal temperature with 12 h light/dark cycle and 30–70% relative humidity. The animals were provided with free access to standard pelleted rodent feed (M/s Gold Mohur Foods and Feeds Ltd., India) and UV purified and filtered water, *ad libitum*. The experimental protocol was approved by the Institutional Animal Ethics Committee of College of Veterinary and Animal Sciences, Kerala Agricultural University. 

### 2.2. Experimental Diets

The composition of the various NCDs and HFDs formulated in the present study was based on AIN-93G rodent diet composition as recommended by the American Institute of Nutrition, [5] with minor modifications in the fat components, namely, soya oil and lard. The different NCDs provided 7 to 12% of the total energy from fat (soya oil) whereas HFDs provided 45% to 60% from fat (soya oil and lard), by substituting energy from carbohydrate. When fat components were changed, care was taken to ensure that experimental diets have a similar nutrient to calorie ratio, as animals will mostly eat for calories and not weight of food [9]. The composition of different NCDs and HFDs tested is illustrated in Table 1. The test diets were fed to rats *ad libitum*.

### 2.3. Experimental Design

Male albino Wistar rats were randomly assigned into seven groups. Group I was fed with standard normal pellet diet (NPD), that is, chow diet. Groups II to IV were fed with customized NCDs I, II, and III while groups V to VII received customized HFDs I, II, and III, respectively. The diets were fed to the experimental rats for a period of 12 weeks *ad libitum*. During the study period, body weight was recorded at fortnightly intervals and body weight gain was calculated. Blood was collected on days 0, 42, and 84 for estimation of serum triglyceride, total cholesterol, and glucose. At the end of the experiment, adipose depots such as epididymal, perirenal, retroperitoneal, and mesenteric fat pads were collected after sacrifice, and the relative weights were calculated. Based upon the findings of the study, the NCD that exhibited close resemblance to normal pellet diet/chow diet was selected as the normal control diet and the one producing metabolic alterations consistently with comparatively lesser fat content among different HFDs was finalized as the high fat diet for induction of metabolic disorders. 

The selected HFD was further evaluated for induction of fatty liver by analyzing terminal total hepatic lipid and hepatic triglyceride levels in comparison to the selected normal control diet, while induction of type 2 diabetes was confirmed in an HFD and low-dose streptozotocin induced diabetes model in Wistar rats. 

### 2.4. Development of HFD/STZ Induced Type 2 Diabetes Model

Type 2 diabetes was induced in rats by feeding HFD (the selected HFD) for 2 weeks, followed by a single intraperitoneal injection of streptozotocin (STZ) at a low dose (35 mg/kg body weight, dissolved in 0.05 M citrate buffer, pH 4.5). The rats with random serum glucose level of ≥300 mg/dL by one week of injection were considered as diabetic [10]. Normal control rats were fed with NCD (the selected NCD) for 2 weeks and on day 14, they were injected with citrate buffer, i.p. Animals from both the groups were maintained for another 6 weeks. Blood was collected fortnightly during the observation period for analyzing the random serum glucose. The terminal blood was collected after overnight fasting and serum glucose and insulin were measured for computing the insulin indices, namely, homeostatic model assessment (HOMA) and quantitative insulin-sensitivity check index (QUICKI) [11] as follows:

(1) HOMA=fasting  insulin(μU/mL)×fasting  glucose(mmol/L)  22.5QUICKI=1[log⁡ fasting  insulin(μU/mL)+log⁡ fasting  glucose(mg/dL) ].

### 2.5. Statistical Analysis

All values are expressed as mean ± SEM and statistical analysis was carried out using statistical package for social science (SPSS). The data were analyzed using one-way ANOVA followed by Bonferroni's post hoc test for multiple group comparison. Student's *t*-test was carried out to determine the significant difference in parameters of studies with NCD-I and HFD-I treatment groups alone. Statistical significance was set at *P* ≤ 0.05.

## 3. Results 

### 3.1. Effect of Experimental Diets on Body Weight and Adiposity

**Figure 1:**
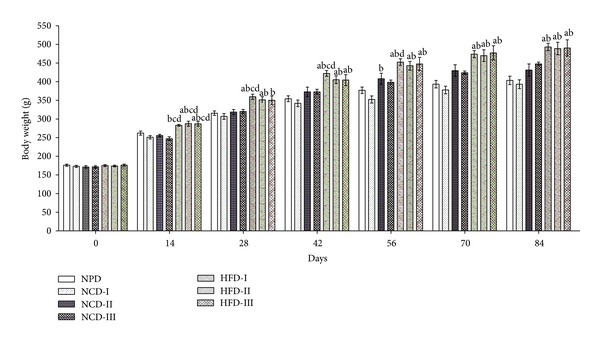
Effect of different experimental diets on body weight in albino Wistar rats. Body weight in normal pellet diet (NPD), normal control diets (NCDs I, II, and III), and high fat diets (HFDs I, II, and III) groups at fortnightly intervals during 12 weeks of study period. Values are expressed as mean ± SEM. ^a^ *P* ≤ 0.05 versus NPD, ^b^ *P* ≤ 0.05 versus NCD-I, ^c^ *P* ≤ 0.05 versus NCD-II, and ^d^ *P* ≤ 0.05 versus NCD-III.

The NPD and NCD groups showed no significant difference in body weight throughout the study period except on day 56 wherein NCD-II showed a significant (*P* ≤ 0.05) increase in body weight as compared to NCD-I. All the high fat diet (HFD) groups exhibited a significant (*P* ≤ 0.05) increase in body weight from day 14 through day 84 as compared to NPD and NCD-I groups except for a nonsignificant increase observed in HFD-I on day 14 and HFD-III on day 28 as compared to NPD group. In addition, the HFD groups showed significant (*P* ≤ 0.05) increase in body weight in comparison to all NCD groups at some of the time intervals (Figure 1).

**Figure 2:**
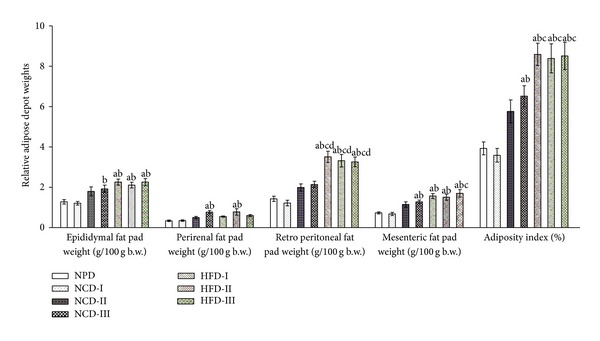
Effect of different experimental diets on adiposity in albino Wistar rats. Adiposity in normal pellet diet (NPD), normal control diets (NCDs I, II, and III), and high fat diets (HFDs I, II, and III) groups after 12 weeks of study period. Values are expressed as mean ± SEM. ^a^ *P* ≤ 0.05 versus NPD, ^b^ *P* ≤ 0.05 versus NCD-I, ^c^ *P* ≤ 0.05 versus NCD-II, and ^d^ *P* ≤ 0.05 versus NCD-III; adiposity index = sum of the fat pads/(body weight-fat pad weight) × 100.

There was no significant difference in relative weights of different fat pads between NPD, NCD-I, and NCD-II groups. However, NCD-III group showed a significant (*P* ≤ 0.05) increase in relative epididymal fat weight in comparison to NCD-I group and a significant (*P* ≤ 0.05) increase in relative perirenal fat and mesenteric fat weights and adiposity index as compared to NPD and NCD-I groups. All the HFD groups showed a significant (*P* ≤ 0.05) increase in relative epididymal and mesenteric fat weights as compared to NPD and NCD-I groups while relative perirenal fat weight was significantly (*P* ≤ 0.05) increased in HFD-II group alone. All HFD groups showed a significant (*P* ≤ 0.05) increase in relative retroperitoneal fat weight as compared to NPD and all the NCD groups, while significant (*P* ≤ 0.05) increase in adiposity index was observed with respect to NPD, NCD-I, and NCD-II groups (Figure 2). 

### 3.2. Effect of Experimental Diets on Biochemical Parameters

There was no significant difference in the level of serum triglyceride, total cholesterol, and glucose among rats of NPD and NCD groups during the study period except for a significant (*P* ≤ 0.05) increase in serum triglyceride observed in NCD-III on day 84 when compared to NCD-I. Meanwhile, all the HFD groups showed significant (*P* ≤ 0.05) increase in serum triglyceride in comparison to NPD and NCD-I groups from day 42 through day 84 of the experiment. In case of serum total cholesterol level, there was a significant (*P* ≤ 0.05) increase in HFD-I and HFD-III groups on day 42 and day 84 in comparison to NPD and all the NCD groups while HFD-II exhibited significant (*P* ≤ 0.05) increase in serum total cholesterol levels as compared to NPD and NCD-II on day 42 and in comparison to NPD and NCD-I on day 84. However, no significant difference was observed in serum glucose levels among rats of all HFD groups as compared to NPD and NCD groups during the study period (Table 2).

### 3.3. Effect of Selected High Fat Diet on Induction of Fatty Liver and Type 2 Diabetes

**Figure 3:**
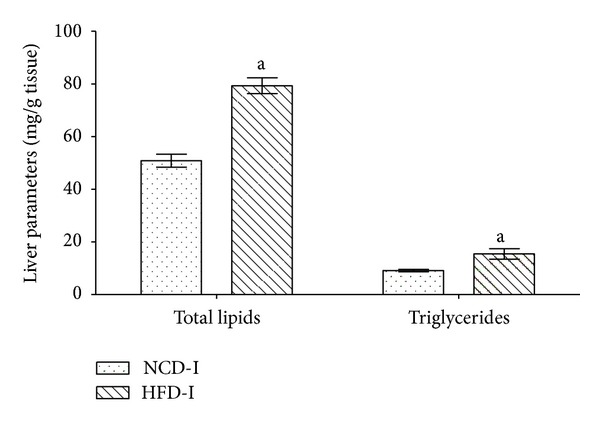
Effect of NCD-I and HFD-I on terminal hepatic lipid parameters in albino Wistar rats. Terminal hepatic lipid parameters in NCD-I and HFD-I fed albino Wistar rats after 12 weeks of study period. Values are expressed as mean ± SEM; *n* = 5. ^a^ *P* ≤ 0.05 versus NCD-I. NCD: normal control diet, HFD: high fat diet.

**Figure 4:**
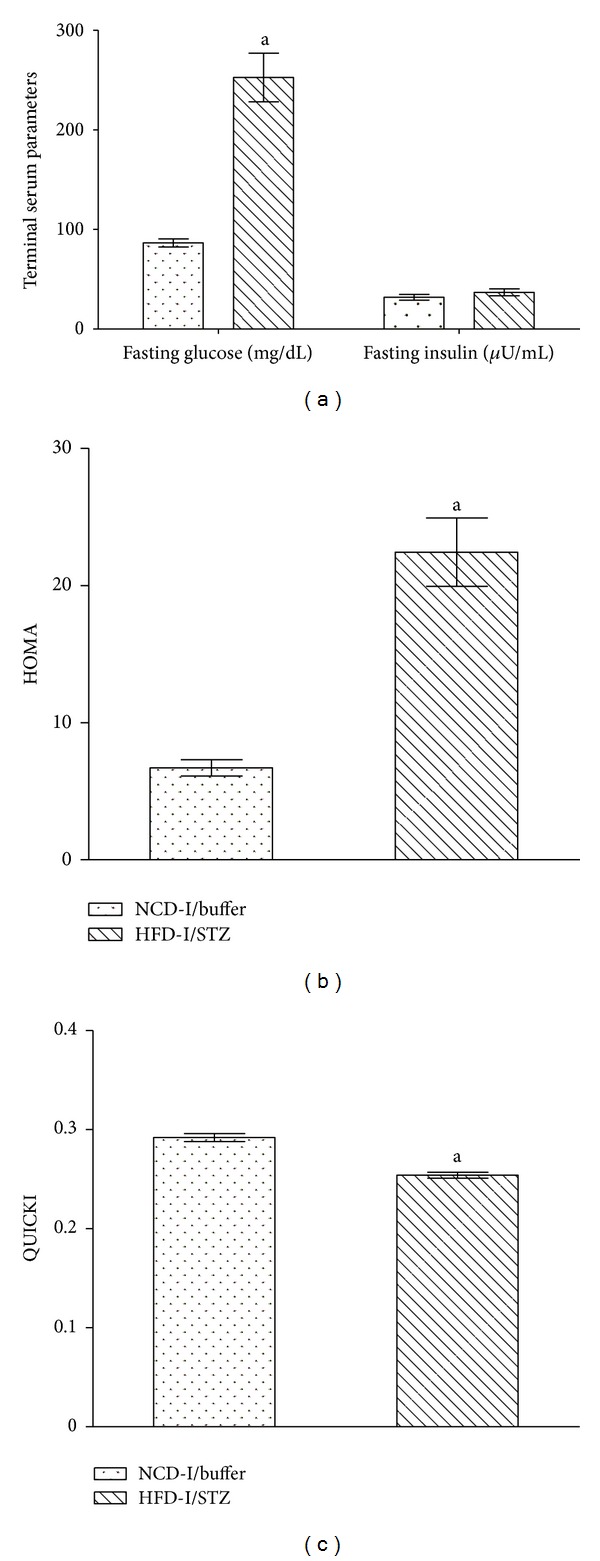
Effect of NCD-I/buffer and HFD-I/STZ treatment on terminal insulin indices in albino Wistar rats. Terminal insulin indices in NCD-I/buffer and HFD-I/STZ treated albino Wistar rats after 6 weeks of induction. Values are expressed as mean ± SEM; *n* = 6. (a) Fasting serum glucose and insulin, (b) HOMA, and (c) QUICKI. ^a^ *P* ≤ 0.05 versus NCD-I/buffer. NCD: normal control diet, HFD: high fat diet, STZ: streptozotocin, HOMA: homeostatic model assessment, QUICKI: quantitative insulin-sensitivity check index.

The selected HFD-I fed rats showed a significant (*P* ≤ 0.05) increase in hepatic total lipids and triglyceride as compared to selected NCD-I fed normal control rats after 12 weeks of study period (Figure 3). In case of HFD/STZ induced type 2 diabetes model in male Wistar rats, HFD-I/STZ treated animals showed significant (*P* ≤ 0.05) increase in HOMA and a significant (*P* ≤ 0.05) decrease in QUICKI in comparison to NCD-I/buffer treated normal control rats. Moreover, HFD-I/STZ treated diabetic rats exhibited a significant (*P* ≤ 0.05) increase in random as well as fasting serum glucose levels, with the serum insulin concentration comparable in absolute terms to the insulin level seen in NCD-I/buffer treated normal control animals (Table 3 and Figure 4).

## 4. Discussion

The present study was conducted with an objective of selecting appropriate normal control and high fat diets for research studies on metabolic disorders, for which different diets were administered for a period of 12 weeks in male Wistar rats and tested for diverse significant effects in the metabolic parameters. The body weight of NCD fed rats from NCD-I and III groups did not differ significantly from NPD fed rats, while NCD-II showed a significant increase in body weight as compared to NCD-I. Similarly, relative weights of different adipose depots did not differ significantly between NPD, NCD-I, and NCD-II groups, although NCD-III group showed a significant increase in relative epididymal fat, perirenal fat, and mesenteric fat weights and adiposity index as compared to other normal diet fed rats. Moreover, normal diet fed rats did not differ significantly in mean serum triglyceride, total cholesterol, and glucose levels among themselves except for a significant increase in serum triglyceride level observed in NCD-III on day 84 as compared to NCD-I. 

In AIN-93G formulation, soybean oil containing two dietary essential fatty acids, the linoleic and linolenic acids, is the major fat component, recommended at a level of 70 g/kg diet. However, the minimal requirements for rats are reported to be 12 g linoleic acid and 2 g *α*-linolenic acid/kg diet which can be offered by soya oil at 30 g/kg of diet as a single source of fat [6]. In the present study, soya oil was therefore used at 30, 40, and 50 g/kg of diet in NCD-I, II, and III, respectively, in order to detect the NCD showing maximum resemblance with the normal chow diet. The present finding thus indicated a close resemblance of the NCD-I (30 g soya oil/kg diet) with the NPD and hence was selected for the subsequent metabolic studies as the normal control diet. 

On the other hand, all the three HFD fed groups exhibited a significant increase in body weight as compared to NPD and NCD-I groups at most of the time intervals after 6 weeks of study period. In addition, the body weight was found to be >20% as compared to normal control group, indicating effective induction of obesity by HFD diet as reported by Lei et al. [12]. Furthermore, all the HFD groups showed a significant increase in relative epididymal fat, mesenteric fat, and retroperitoneal fat weights and adiposity index as compared to NPD and NCD-I groups. In case of biochemical parameters, all the HFD groups showed a significant increase in serum triglyceride and cholesterol levels in comparison to normal diet fed rats. Conversely, there was no significant difference in serum glucose levels amongst rats of all HFD groups during the study period. Thus, all the three HFDs taken for study produced almost comparable body weight gain; albeit a relatively higher level of adiposity index, serum TG and TC concentrations were observed in HFD-I and HFD-III fed rats as compared to normal diet fed rats, indicating effective establishment of diet-induced animal models for obesity and dyslipidemia. 

However, while studying the effects of a drug, nutraceutical, or gene mutation on obesity, it might be more difficult to prevent or reverse the effects of a very high fat diet (VHFD) that contains greater than 50% kcal fat, though it might be possible with a high fat diet (HFD) which is supposed to contain 30–50% of the calories coming from fat [2]. Therefore, in the present study, despite HFD-I (45% kcal fat, 21.3% kcal protein, and 33.7% kcal carbohydrate) and HFD-III (60% kcal fat, 20.8% kcal protein, and 19.2% kcal carbohydrate) producing metabolic alterations consistently, HFD-I containing lesser fat was recommended for further studies of diet-induced metabolic diseases in Wistar rats. 

There is extensive literature characterizing responses to high fat feeding in rodents, and the findings of the present study are in corroboration with previous reports [13–16]. HFDs produce a consistent and significant increase in body weight, adipocyte hypertrophy and hyperplasia, dyslipidemia, and insulin resistance, depending on fat in the diet, duration of feeding, and strain of the experimental animal [1, 15, 17]. Consumption of energy rich HFD leads to obesity because it facilitates the development of a positive energy balance. Moreover, diet rich in saturated fatty acids like lard decreases diet-induced thermogenesis by a decline in sympathetic activity in brown adipose tissue [18]. The imbalance in energy homeostasis thus developed in HFD fed rats in the study would have obviously led to increase in body weight, majorly by an increase in visceral fat deposition.

The pandemic of obesity predicts major increases in the incidence of the metabolic syndrome, characterized by a constellation of disorders, including insulin resistance, dyslipidemia, impaired glucose tolerance, cardiovascular disease, and NAFLD that precedes the development of type 2 diabetes [19]. NAFLD associated with obesity and hyperlipidemia encompasses a spectrum of histopathology, ranging from steatosis to cirrhosis. Steatosis may be progressed into end-stage liver disease, if optimal treatment has not been established [20]. Feeding HFD to rats is a widely-used method of establishing a NAFLD model [21, 22]. In the present study after 6 weeks of feeding, total hepatic lipids and triglyceride were increased significantly in HFD-I fed rats indicating effective induction of fatty liver, specifically NAFLD as reported in earlier studies [23]. Enhanced delivery of fatty acid from enlarged visceral adipose tissue to the liver leads to reduced hepatic insulin clearance, stimulates hepatic gluconeogenesis and hepatic triglyceride synthesis, and also impairs insulin suppression of hepatic glucose output [24]. In liver, hyperinsulinemia induces sterol regulatory element–binding protein-1c (SREBP-1c) expression, leading to the transcriptional activation of all lipogenic genes [25]. 

In HFD/STZ model, HFD initiates a state of insulin resistance followed by the addition of low-dose STZ that induces moderate impairment of insulin secretion, which is a characteristic of the later stage of human type 2 diabetes mellitus [11]. HOMA and QUICKI are the two convenient tests for diagnosis of insulin resistance and its metabolic manifestations and can be adopted for epidemiological studies as well as clinical practice [26]. The HFD/STZ treated animals in the present study showed significant increase in HOMA, the insulin resistance index, and a significant decrease in QUICKI, insulin-sensitivity index, in comparison to normal control rats, which clearly indicated the development of insulin resistance in the experimental diabetic rats. Moreover, the significant increase in random as well as fasting serum glucose levels observed in HFD/STZ treated diabetic rats, along with serum insulin concentration comparable in absolute terms to the insulin level of normal control animals, was analogous to the previously published reports, [27–29] indicating the effective induction of type 2 diabetes. 

Insulin resistance is a characteristic metabolic defect that precedes overt *β*-cell dysfunction and is primarily associated with resistance to insulin-mediated glucose disposal at the periphery and compensatory hyperinsulinemia. In the course of time, the *β*-cell function gets impaired leading to deterioration in glucose homeostasis and the development of impaired glucose tolerance and frank diabetes. Therefore, only a relative insulin deficiency exists with type 2 diabetes as the day-long circulating insulin concentrations in patients are almost comparable or slightly elevated in absolute terms to the values in normal individuals [10]. Impaired muscle glucose uptake is the main cause of postprandial hyperglycemia whereas overproduction of glucose by the liver and glucagon dysregulation are the main causes of fasting hyperglycemia. These defects that produce fasting hyperglycemia further contribute to postprandial hyperglycemia. Moreover, impaired suppression of lipolysis results in higher circulating plasma FFA levels which in turn may contribute to both muscle insulin resistance and overproduction of glucose by the liver [30].

Based on the present observations, an appreciation of the current animal model can be made by considering the following facts. Although different high fat diets are used for induction of metabolic disorders, the present diet is unique in view of its composition, which was based on AIN-93 recommendations and such that both NCD and HFD possessed same nutrient to calorie ratio, providing an ideal base for comparison of diets. Furthermore, the selected HFD effectively induced obesity and associated metabolic disorders such as dyslipidemia, insulin resistance, and NAFLD. With respect to HFD/STZ induced type 2 diabetic models, there are different combination strategies of HFD and STZ treatment with pros and cons for each combination. It was Srinivasan et al. [10] who has proposed the HFD/STZ combination rat model of 2 weeks' HFD administration followed by STZ administration at a lower dosage of 35 mg/kg, which has been used in the present study and is claimed to mimic the natural history and metabolic characteristics of the common type 2 diabetes in humans. Although, the model was unique, Srinivasan et al. [10] have used HFD as a mix of chow diets and fat components like lard. On the contrary, in the present study, same HFD/STZ treatment schedule was pursued with semipurified HFD having same nutrient to calorie ratio with that of NCD and the study revealed comparable results in type 2 diabetes induction. 

## 5. Conclusion

The current global rise in the incidence of obesity and type 2 diabetes, majorly posed by western-style diet, has created an urgent need to identify innovative diet-induced animal models for metabolic syndrome. To establish appropriate semipurified normal control diet (NCD) and high fat diet (HFD), for research studies on metabolic disorders, different diets were formulated in the present study, based on American Institute of Nutrition (AIN-93) rodent diet composition with minor modifications in the fat component and tested in male Wistar rats. The results of the study clearly suggest that normal control diet-I (containing 7.1% kcal fat with 30 g soya oil/kg diet) showing close resemblance with normal pellet diet/chow diet and high fat diet-I (containing 45% kcal fat with 37.5 g soya oil and 200 g lard/kg diet) producing metabolic alterations consistently with lesser fat content can be recommended for research studies on diet-induced metabolic disorders in albino Wistar rats.

## Figures and Tables

**Table 1:** Composition of experimental normal control diets (NCDs) and high fat diets (HFDs).

Ingredient	Normal control diets						High fat diets					
	NCD-I		NCD-II		NCD-III		HFD-I		HFD-II		HFD-III	
	g	kcal	g	kcal	g	kcal	g	kcal	g	kcal	g	kcal
Casein	200	800	200	800	200	800	250.1	1000	244.4	978	271.3	1085
L-Cystine	3	12	3	12	3	12	3.75	15	3.67	15	4.07	16
Soybean oil	30	270	40	360	50	450	37.5	338	61.1	550	67.8	610
Lard	0	0	0	0	0	0	200.3	1803	177.4	1597	285.3	2568
Cornstarch	438.59	1754	428.59	1714	418.59	1674	97.65	391	112.28	449	52.53	210
Maltodextrin	132	528	132	528	132	528	165.1	660	161.3	645	52.53	210
Sucrose	100	400	100	400	100	400	125	500	122	488	135.6	542
AIN-93 vitamin mix	10	40	10	40	10	40	12.5	50	12.2	49	13.6	54
AIN-93G mineral mix	35	0	35	0	35	0	43.8	0	42.8	0	47.5	0
Choline chloride	1.4	0	1.4	0	1.4	0	1.75	0	1.7	0	1.9	0
Cellulose	50	0	50	0	50	0	62.5	0	61.1	0	67.8	0
t-BHQ	0.01	0	0.01	0	0.01	0	0.05	0	0.05	0	0.07	0
Total	1000	3804	1000	3854	1000	3904	1000	4757	1000	4771	1000	5295

Formula	NCD-I		NCD-II		NCD-III		HFD-I		HFD-II		HFD-III	
	g%	kcal%	g%	kcal%	g%	kcal%	g%	kcal%	g%	kcal%	g%	kcal%

Protein	20.3	21.3	20.3	21.1	20.3	20.8	25.4	21.3	24.8	20.8	27.5	20.8
Carbohydrate	68.1	71.6	67.1	69.6	66.1	67.7	40.0	33.7	40.8	34.2	25.4	19.2
Fat	3	7.1	4	9.3	5	11.5	24	45.0	24	45.0	35	60.0
Total		100		100		100		100		100		100
kcal/g	3.8		3.9		3.9		4.8		4.8		5.3	

**Table 2:** Effect of different experimental diets on biochemical parameters in male Wistar rats.

Treatment groups	Serum triglyceride (mg/dL)			Serum total cholesterol (mg/dL)			Serum glucose (mg/dL)		
	Day 0	Day 42	Day 84	Day 0	Day 42	Day 84	Day 0	Day 42	Day 84
I NPD	91.18 ± 7.94	120.55 ± 9.45	148.24 ± 5.38	67.54 ± 2.31	60.14 ± 2.31	63.51 ± 2.56	85.85 ± 2.92	92.03 ± 3.70	102.66 ± 8.35
II NCD-I	99.80 ± 6.97	116.26 ± 7.53	126.19 ± 9.63	64.99 ± 2.71	66.26 ± 4.35	64.29 ± 4.49	88.49 ± 3.94	99.40 ± 5.36	100.60 ± 3.92
III NCD-II	98.78 ± 8.67	148.87 ± 9.21	177.90 ± 14.51	62.57 ± 2.14	64.43 ± 3.39	66.11 ± 4.23	92.87 ± 2.82	102.20 ± 5.76	102.70 ± 2.94
IV NCD-III	98.13 ± 4.74	154.12 ± 8.80	188.92 ± 13.20^b^	66.41 ± 2.11	68.55 ± 2.24	68.53 ± 3.97	85.34 ± 3.09	101.78 ± 3.19	105.55 ± 4.64
V HFD-I	91.88 ± 8.54	193.68 ± 11.91^ab^	248.97 ± 19.26^ab^	67.42 ± 4.80	83.41 ± 3.02^abcd^	91.47 ± 1.89^abcd^	90.24 ± 4.08	109.09 ± 5.49	119.24 ± 11.42
VI HFD-II	99.49 ± 5.84	182.85 ± 13.28^ab^	238.66 ± 21.65^ab^	69.07 ± 2.46	80.48 ± 3.01^ac^	80.34 ± 1.75^ab^	87.56 ± 3.10	106.54 ± 8.39	115.84 ± 9.30
VII HFD-III	97.92 ± 5.10	188.13 ± 21.28^ab^	279.21 ± 31.11^abc^	68.54 ± 2.70	87.09 ± 4.03^abcd^	89.00±4.03^abcd^	93.46 ± 2.32	116.35 ± 6.45	125.34 ± 9.27

Values are expressed as mean ± SEM; *n* = 8.

^a^ *P* ≤ 0.05 versus NPD, ^b^ *P* ≤ 0.05 versus NCD-I, ^c^ *P* ≤ 0.05 versus NCD-II, and ^d^ *P* ≤ 0.05 versus NCD-III.

NPD: normal pellet diet, NCD: normal control diet, and HFD: high fat diet.

**Table 3:** Effect of NCD-I/buffer and HFD-I/STZ treatment on serum glucose level in albino Wistar rats.

Treatment groups	Serum glucose (mg/dL)					
	Induction period			Treatment period		
	Day 0	Day 14	Day 21	Day 14	Day 28	Day 42
I NCD-I/buffer	107.16 ± 7.51	101.80 ± 8.01	101.35 ± 5.27	105.59 ± 10.79	103.91 ± 5.11	106.26 ± 9.62
II HFD-I/STZ	101.40 ± 5.49	104.00 ± 6.14	529.36 ± 34.10^a^	527.50 ± 29.68^a^	506.17 ± 38.24^a^	541.94 ± 38.58^a^

Values are expressed as mean ± SEM; *n* = 6–9.

^a^ *P* ≤ 0.05 versus NCD-I/buffer.

NCD: normal control diet, HFD: high fat diet, STZ: streptozotocin.
